# Identification and characterisation of carbapenem-resistant *Streptococcus nidrosiense* sp. nov. isolated from blood culture

**DOI:** 10.1016/j.nmni.2024.101473

**Published:** 2024-08-27

**Authors:** Torunn Gresdal Rønning, Camilla Olaisen, Christina Gabrielsen Ås, Jan Egil Afset, Maria Schei Haugan

**Affiliations:** a Department of Medical Microbiology, St. Olavs Hospital, Trondheim University Hospital, Trondheim, Norway; b Department of Clinical and Molecular Medicine, Norwegian University of Science and Technology, Trondheim, Norway

**Keywords:** *Streptococcus nidrosiensis*, Viridans streptococci, Carbapenem resistance

## Abstract

**Background:**

This study aimed to investigate a highly resistant strain of *Streptococcus* sp. isolated from a patient with bloodstream infection and determine its taxonomic classification.

**Methods:**

The strain was isolated from blood culture from a 65-year-old male patient admitted to St. Olavs University hospital, Trondheim, Norway, in 2023. Antimicrobial susceptibility testing as well as phenotypic and biochemical characterization were performed. Whole genome sequencing was conducted and genomic comparison to *Streptococcus* type strains was carried out.

**Results:**

The strain was initially identified as *Streptococcus mitis*/*oralis* but showed significant genetic differences, suggesting that it belonged to an undescribed species within the *Streptococcus* genus. Phenotypic and biochemical characterization identified the strain as a non-motile, facultative anaerobic bacterium with α-hemolysis. Antimicrobial susceptibility testing showed resistance to all beta-lactams tested. Genomic analyses confirmed the classification of the strain as a novel species, which was designated *Streptococcus nidrosiense*.

**Conclusion:**

This study combines conventional phenotypic tests with whole genome sequencing for accurate taxonomic classification of a bacterial strain isolated from blood culture. The identification of a novel species within the *Streptococcus* genus contributes to the understanding of microbial diversity and antibiotic resistance of the *Streptococcus* genus in clinical settings.

## Highlights

• We isolated a novel strain of *Streptococcus* sp. from a patient with BSI.
• Unusual pattern of antibiotic resistance, including carbapenem-resistance.
• Carbapenemase antibiotic resistance in VGS is a concerning healthcare issue.
• Accurate taxonomic classification by phenotypic and genotypic analysis.
• We designate the novel species *Streptococcus nidrosiense*.

## 1 Introduction

The viridans group streptococci (VGS) encompass a diverse array of catalase-negative, Gram-positive cocci that are capable of serving as commensals within the human body. These organisms have the ability to colonize various anatomical sites such as the gastrointestinal and genitourinary tracts, in addition to the oral mucosa [1]. However, the VGS can also be human pathogens, especially in immunocompromised individuals and neonates, causing invasive infections such as endocarditis, meningitis, sepsis and pneumonia [2]. Taxonomy of the VGS group has been inconsistent and even controversial, due to large variation in hemolytic activity, differences in substrate metabolism as well as high level of sequence homology between 16S rRNA genes within the group. There are however currently more than 30 recognized species of VGS [1,3], which are categorized into six significant groups: the *S. mutans* group, the *S. salivarius* group, the *S. anginosus* group, the *S. mitis* group, the *S. sanguinis* group, and the *S. bovis* group [4]. Recently, whole genome sequencing-based methods, including Average Nucleotide Identity (ANI) [5,6] and digital DNA–DNA Hybridization (dDDH), have emerged as effective tools for the classification of a large number of species, particularly when conventional biochemical tests are insufficient to accurately assign a strain to its appropriate species designation [7]. These methods have thus been able to distinguish more clearly between the VGS, as well as aid in the designation of novel species [8,9].

In 2023, a highly resistant strain (hereafter termed SO-23-1) of *Streptococcus* sp. was isolated from blood culture from a patient who suffered from a severe polymicrobial bloodstream infection with fatal outcome. Initially, routine analyses identified the strain as *Streptococcus oralis*. Due to its high level of beta-lactam resistance, which is unusual for streptococci, whole-genome sequencing was conducted for further investigation. Data analysis revealed that the isolated bacterial strain was genetically closely related to *S. oralis*, but with significant differences, suggesting that the strain belongs to a novel bacterial species within the *Streptococcus* genus. The aim of this study was to characterize and determine the taxonomic classification of this strain.

## 2 Materials and methods

### 2.1 Case presentation and strain identification

Strain SO-23-1 was isolated from blood culture from a 65-year-old male patient admitted to St. Olavs University hospital, Trondheim, Norway, in 2023. The patient had lung cancer, adenocarcinoma stage T4N0M0, with extensive lesions including tumor growth into the mediastinum and a bronchopleural fistula. He was initially admitted to the hospital due to community-acquired pneumonia with pleural effusion. Initial culture of sputum and pleural fluid both showed polymicrobial growth of a mixture of aerobic and anaerobic bacteria, likely belonging to the oral microbiota. The patient's condition was later, during hospital admission, complicated by pneumothorax, pleural empyema and bacteraemia. Strain SO-23-1 was isolated after two months hospitalisation, together with *Rothia mucilaginosa* and *Lacticaseibacillus rhamnosus*, from blood culture (BACTEC Plus/F Aerobic) after overnight incubation at 35 °C in a Becton Dickinson BACTEC FX blood culture system. Repeated bacterial culture of the pleural fluid showed similar polymicrobial growth of what was considered a mixture of upper airway bacteria, and *Pneumocystis jirovecii* DNA was detected in a sputum sample. Prior to the isolation of bacteria from blood culture, the patient had received empirical treatment with meropenem for several weeks without significant clinical improvement. Due to the lack of treatment effect and the isolation of *streptococci* in blood culture, antimicrobial treatment was changed to piperacillin-tazobactam. Two days later, treatment was further changed to clindamycin, as well as metronidazole, due to detection of unusual beta-lactam resistance of the *Streptococcus* species. In addition, trimethoprim-sulfamethoxazole was given to cover infection with *Pneumocystis jirovecii*. Due to lack of treatment response and advanced stage of cancer disease, active treatment was discontinued after 24 h and the patient died the following day.

Identification of the bacterial strain was performed using matrix-assisted laser-desorption/ionization time-of-flight mass spectrometry (MALDI-ToF MS) [10], with a MALDI Biotyper Sirius instrument and the Bruker Daltonics database V12.0.0.0_10833–11897 on colonies obtained from sheep blood agar COLS+ (Oxoid). Log (score) values above 2.0 were accepted as probable species-level identification, while scores between 1.7 and 1.9 were accepted as probable genus-level identification [11]. Subsequent investigations involved ten replicates of the SO-23-1 strain using the same MALDI-ToF MS technology.

### 2.2 Antimicrobial susceptibility testing

Antimicrobial susceptibility testing was initially performed using the EUCAST standardised disc diffusion method, followed by MIC confirmation by broth microdilution (Sensititre™ Streptococcus STP6F and Sensititre™ Gram Positive GPALL1, Thermo Scientific) according to EUCAST guidelines [12]. *In silico* detection of antibiotic resistance genes was performed using AMRFinder Plus [13], and possible mutations in genes encoding penicillin-binding proteins (PBPs) were analysed using Geneious Prime v2023.0.4 software (Biomatters).

### 2.3 Phenotypical and biochemical characterisation

The culture was grown on sheep blood agar COLS+ in aerobic and anaerobic atmosphere with 24- and 48-h incubation at 35 °C. The strain was also cultured in tryptic soy broth (TSB) (SIGMA-ALDRICH) for 24 h at 35 °C, in normal atmosphere and in 5 % CO_2_. Light microscopy of wet mount and fixed Gram stained [14] liquid culture was performed to investigate motility and morphology of the bacterium.

Catalase- (bioMérieux) and coagulase-tests (in-house plasma) [15] were performed as previously described.

A NaCl tolerance test [16] was performed by preparing a dilution series of NaCl in TSB, with the following NaCl concentrations: 0.5, 1.0, 2.0, 3.0, 4.0, 5.0, 6.0, 6.5 and 7.0 %. Briefly, 1–3 bacterial colonies were inoculated in triplicate in TSB with added NaCl, and observation of visible turbidity after 24 h of incubation at 35 °C in 5 % CO_2_ atmosphere was considered a positive result.

Biochemical characteristics of the strain SO-23-1 were determined by API 20 STREP according to the manufacturer's instructions (bioMérieux).

### 2.4 DNA extraction and whole genome sequencing

Bacterial colonies were dissolved in 200 μL TE buffer containing 1.5 mg/mL lysozyme, 0.5 mg/mL proteinase K (Qiagen) and 250 U/mL mutanolysin (Merck), before 15 min incubation at 37 °C, 15 min at 65 °C, and addition of 100 mg/mL RNaseA. Genomic DNA was subsequently extracted using the EZ1 DNA Tissue kit (Qiagen) with an EZ1 Advanced XL extractor (Qiagen). A short-read sequencing library was prepared using Illumina DNA prep, and sequenced using the MiSeq reagent kit v3 (2x300 bp) on a MiSeq Instrument (Illumina). A long-read sequencing library was prepared using the rapid sequencing kit SQK-RAD004 and sequenced on a flongle flow cell (FLO-FLG001, R.9.4.1) using a MinION Mk1b instrument (Oxford Nanopore Technologies) [17]. Long-read data were basecalled using Guppy v5.1.15 (Oxford Nanopore Technologies) and assembled using the Flye assembler v2.7 [18]. The assembly was furthermore circulated with circlator v1.5.5 [19] and polished with long read data using using Racon v1.4.20 [20], before polishing with short-read data using Pilon v1.23 [21].

### 2.5 Genomic annotation and comparison

Reference genomes for *Streptococcus mitis* group type strains were downloaded from RefSeq Database (NCBI) and annotated using Prokka v1.14.6 [22]. Complete nucleotide sequences of housekeeping genes 16S rDNA, 23S rDNA, *rpoB*, *sodA* and *gyrA* were extracted, aligned with MUSCLE v5.1 and Neighbour-joining phylogenies inferred with the Tamura-Nei distance model using the Geneious treebuilder in Geneious Prime v2023.0.4 (Biomatters). Pangenome analysis was performed using Roary v3.13.0 [23] with identity treshold 70 %, and a core genome phylogeny inferred using FastTree [24] with the GTR substitution model. Calculation of the percentage of conserved proteins (POCP) was performed using the POCP Nextflow pipeline [25,26]. The genome of strain SO-23-1 was taxonomically classified and closely related strains identified using the Type Strain Genome Server (TYGS) pipeline as well as the Genome Taxonomy Database Toolkit (GTDB-tk). Finally, BLAST-based Average Nuclotide Identity (ANIb) was calculated using Jspecies WS [5] and digital DNA-DNA hybridisation was performed using the Genome-to-Genome Distance Calculator (GGCD) webserver [27], with the recommended formula 2 (sum of all identities/HSP length) used for score calculation.

The genome sequence data were registered under GenBank BioProject PRJNA1101760 (accession numbers: **CP152419** (whole genome sequence), **PP761413**, **PP761414**, **PP761415**, **PP761416** (16S rRNA sequences)).

## 3 Results

### 3.1 Phenotypic and biochemical characterisation

**Fig. 1:**
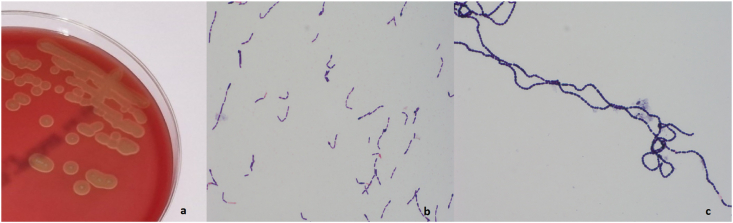
**a**) Growth of *Streptococcus* sp. strain SO-23-1 on sheep blood agar. Image was captured after 48h growth in CO_2_ atmosphere, using backlight. **b**) Gram-stain microscopy of the strain from culture in TSB after 24h incubation in CO_2_ atmosphere, and **c**) after 24h in normal atmosphere. Images b) and c) were captured with Olympus cellSens Entry software version 2.3 with 1000 X amplification on an Olympus BX43 microscope with an Olympus DP26 digital camera.

SO-23-1 exibited facultative anaerobic growth with α-haemolysis on sheep blood agar (Fig. 1a). Here, the colonies were dry and adherent. Both coagulase and catalase tests were negative. The isolate was identified by MALDI-ToF MS as *S. mitis/oralis* from culture grown on sheep blood agar. Further testing by MALDI-ToF MS identified strain SO-23-1 as *S. mitis/oralis* in 9 out of 10 replicates with score values ranging from 1.84 to 2.14 (median 1.98) and in one replicate as *S. infantis* with score value 1.95, all members of the *S. mitis* group.

The bacterium was found to be Gram-positive and non-motile. Morphologically, the bacteria appeared as rod-shaped cocci in chains with a diphteroides-like appearance (Fig. 1b) after incubation with CO_2_. However, after incubation in normal atmosphere, the bacterial cells showed a more characteristic viridans streptococci apperance, with cocci in long chains (Fig. 1c). This is similar to how *S. mutans* can display cellular dimorphism as cocci under neutral or basic growth conditions, and as short rods or diphteroid appearance in acidic culture media [28,29].

The SO-23-1-strain grew well in TSB supplemented with NaCl concentrations ranging from 0.5 % to 3 %. Scarce growth was observed when the NaCl concentration in TSB was increased to 4.0 %. No growth was observed in TSB containing 5.0 % NaCl or higher.

By interpretation of the API 20 STREP test, SO-23-1 was identified as *Streptococcus intermedius* (91.20 % similarity). The strain had positive biochemical reactions towards the substrates ESC, βGAL, PAL, LAP, ADH (negative after 4 h, positive after 24 h), as well as LAC and GAL in the API 20 STREP test. Tests were negative towards substrates HIP, PYRA, αGAL, βGUR, RIB, ARA, SOR, TRE, RAF, AMD and GLYG. The results for VP and MAN were inconclusive (Table 1). Furthermore, type strains for *Streptococcus oralis*, subsp. *oralis* (CCUG 13229T), *Streptococcus oralis*, subsp. *dentisani* (CCUG 66492T), *Streptococcus oralis*, subsp. *tigurinus* (CCUG 66514T), *Streptococcus mitis* (CCUG 31611T), *Streptococcus pneumoniae* (CCUG 33638), *Streptococcus pseudopneumoniae* (CCUG 49455T), *Streptococcus infantis* (CCUG 39817T) and *Streptococcus peroris* (CCUG 39814T) were tested in parallel. However, results were inconclusive, and varied between replicates. Most strains were misidentified in the API database (results not shown). Alpha haemolytic streptococci are known to be difficult to identify using biochemical tests such as the API system [30].

**Table 1:** API 20 Strep results for the SO-23-1 strain.

Test	Active ingredient	Reaction/Enzyme	Result
VP	sodium pyruvate	acetoin production (Voges Proskauer)	Inconclusive
HIP	hippuric acid	hydrolysis (HIPpuric acid	Negative
ESC	esculin ferric citrate	β-glucosidase hydrolysis (ESCulin)	Positive
PYRA	pyroglutamic acid-β-naphthylamide	PYRrolidonyl Arylamidase	Negative
αGAL	6-bromo-2-naphtyl-αD-galactopyranoside	α-GALactosidase	Negative
βGUR	naphthol ASBI-glucuronic acid	β-GlUcuRonisidase	Negative
βGAL	2-naphtyl-βD-galactopyranoside	β-GALactosidase	Positive
PAL	2-naphthyl phosphate	ALKaline Phosphatase	Positive
LAP	L-laucine-β-naphthylamide	Leucine AminoPeptidase	Positive
ADH	L-arginine	Arginine DiHydrogenase	Negative after 4h, positive after 24h
RIB	D-ribose	acidification (RIBose)	Negative
ARA	L-arabinose	acidification (ARAbinose)	Negative
MAN	D-mannitol	acidification (MANnitol)	Inconclusive
SOR	D-sorbitol	acidification (SORbitol)	Negative
LAC	D-lactose	acidification (LACtose)	Positive
TRE	D-trehalose	acidification (TREhalose)	Negative
INU	inulin	acidification (INUlin)	Negative
RAF	D-raffinose	acidification (RAFfinose)	Negative
AMD starch	starch	acidification (AmiDon)	Negative
GLYG	glycogen	acidification (GLYcoGen)	Negative

### 3.2 Antimicrobial susceptibility and detection of resistance genes

Strain SO-23-1 showed phenotypic resistance to all beta-lactams tested, including penicillins, cephalosporins and carbapenems (Table 2).

**Table 2:** Results from antimicrobial susceptibility testing of strain SO-23-1. SIR categories were determined using the EUCAST breakpoints for the viridans group streptococci (VGS). ND: breakpoints not defined by EUCAST. HLAR: high-level aminoglycoside resistance. MLSB: macrolide-lincosamide-streptogramin B. *Inferred from ampicillin.

Antibiotic	MIC (μg/mL)	Interpretation (EUCAST)
Benzylpenicillin	>4	Resistant
Ampicillin	>8	Resistant
Amoxicillin-clavulanate	16	Resistant
Piperacillin-tazobactam	–	Resistant*
Ceftriaxone	>2	Resistant
Cefuroxime - parenteral	>4	Resistant
Cefepime	4	Resistant
Cefotaxime	>4	Resistant
Meropenem	>2	Resistant
Ertapenem	>4	Resistant
Moxifloxacin	≤1	ND
Levofloxacin	1	ND
Gentamicin	8	Resistant
Gentamicin HLAR	≤500	Negative
Vancomycin	1	Susceptible
Clindamycin	≤0.125	Susceptible
Erythromycin	1	ND
Azithromycin	≤0.25	ND
Tetracycline	≤1	ND
Tigecycline	≤0.015	ND
Linezolid	0.5	ND
Trimethoprim-sulfamethoxazole	≤0.5	ND
Daptomycin	0.5	ND
Chloramphenicol	2	ND
Rifampicin	2	ND
Inducible MLSB resistance	–	Negative

No known beta-lactamase genes were detected in the strain, but the intrinsic *pbp2b*-gene (Table 3) was reported by AMRFinder Plus as a probable cause of beta-lactam resistance. The *pbp2b*-gene in strain SO-23-1 contains the previously described T446A- and E476G-amino acid substitutions that are commonly found in clinical penicillin-resistant streptococci [31]. Furthermore, the SO-23-1 strain had multiple mutations in *pbp2b* shown to confer meropenem resistance in *S. pneumoniae*: V225I, S412P, N422Y, T426K, Q427L, S473T, S480A, G497P, N606D, L609T, A619G, N659K, G660N, S664A [32]. Mutations associated with meropenem resistance in *S. pneumoniae* were also found in *pbp1a* (P4Q, L9I, I10A, V21F, N58S, Q61E) and *pbp2x* (A347S) [32]. Two additional resistance genes were detected in strain SO-23-1, *mrs(D)* and *mef(A)* (Table 3), encoding a macrolide efflux transport system described in different *Streptococci* including *S. oralis* [33]. Phenotypic macrolide resistance was however not observed. Strain SO-23-1 did, as reported for other streptococci [34], display intrinsic low-level resistance to gentamicin (Table 2).

**Table 3:** Antibiotic resistance genes detected in strain SO-23-1. Results obtained from AMRFinder plus.

Gene symbol	Sequence name	Antibiotic resistance class	Antibiotic resistance subclass	% Coverage of reference sequence	% Identity to reference sequence	Accession number of closest sequence
*pbp2b*	*Streptococcus pneumoniae* beta-lactam resistant PBP2B	Beta-lactam	Beta-lactam	100.00	90.29	WP_001224884.1
*mef(A)*	macrolide efflux MFS transporter Mef(A)	Macrolide	Erytromycin	100.00	100.00	WP_000417519.1
*msr(D)*	ABC-F type ribosomal protection protein Msr(D)	Macrolide	Erytromycin	100.00	100.00	WP_000420313.1

### 3.3 Bioinformatic analyses

**Fig. 2:**
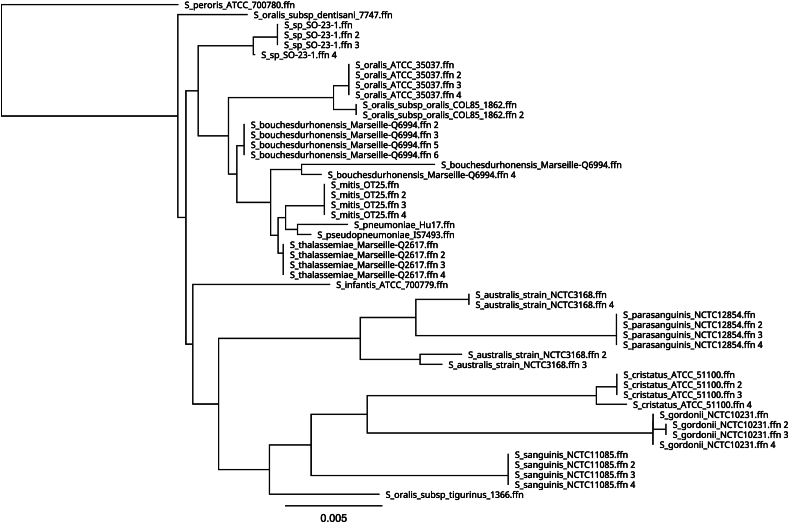
Sequence similarity matrix of 16S rRNA genes of different *Streptococcus* species compared to the four 16S rRNA genes of strain SO-23-1. Scale represents substitutions per site.

While strain SO-23-1 was initially identified as *Streptococcus mitis/oralis* by MALDI-ToF MS, it only shared 98.1–99.2 % sequence similarity of the 16S rRNA gene with *S. oralis* type strains, the highest similarity being to *S. oralis* subsp. dentisani 7747T (Fig. 2). The closest matching *Streptococcus* type strain based on 16S rRNA gene homology was the newly described species *S. bouchesdurhonensis* Marseille-Q6994, with 99.2–99.4 % similarity. However, it has been established that the 16S rRNA gene is insufficient for discrimination between closely related species within the *Streptococcus* genus [35]. Phylogenies of other genes including 23S rDNA, *rpoB*, *sodA* and *gyrA*, similarly showed that the strain clustered separately from other *Streptococcus* reference genomes (results not shown).

The complete genome of strain SO-23-1 consisted of a circular chromosome of 2,018,446 bp with a GC content of 39.7 %. It encoded 1838 protein-coding genes, as well as four rRNA operons and 59 tRNA genes. The SO-23-1 strain had two known virulence genes, *pavA* and *psaA*, which encode a fibronectin-binding protein and a lipoprotein component of an Mn2+ transporter, respectively, both previously described in *S. pneumoniae* [36,37].

**Fig. 3:**
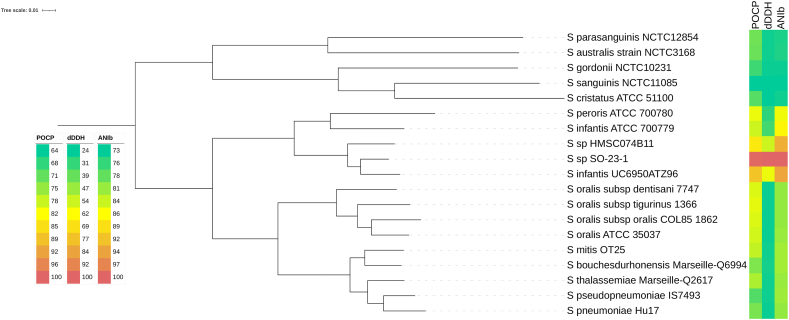
Core genome phylogeny, annotated with results from ANIb, dDDH and POCP analyses of strain SO-23-1 compared to the genetically closest type strains of the *Streptococcus mitis* group. Coloured legend showing lowest (green) to highest (red) percent similarity. Figure made using iTOL [38]. (For interpretation of the references to colour in this figure legend, the reader is referred to the Web version of this article.)

Comparisons between the genome of strain SO-23-1 to those of type strains of the *Streptococcus mitis* group showed >50 % percentage of protein homology, confirming that the strain indeed belonged to the genus *Streptococcus*. Pangenome analysis and core genome phylogeny (Fig. 3) initially identified *S. infantis* ATCC 700779 and *S. peroris* ATCC 700780 as the most closely related type strains, however with protein homology of only 78.9 and 81.1 % accordingly.

Average Nucleotide Indentity (ANI) analysis within GTDB identified the strain *Streptococcus infantis* UC6950A as the best match, notably with taxonomy status marked as « inconclusive». Another close match identified by ANI was *Streptococcus* sp. HMSC074B11, which was taxonomically classified only to genus level. Furthermore, as shown in Fig. 3, the ANIb distances were below the commonly used treshold for within-species designation of 95 %.

There were identified no good matches by digital DNA-DNA hybridisation (dDDH) in the TYGS database, the highest match being *S. infantis* ATCC 700779 with a dDDH score of 34.3 %. Including the closest strains by ANI distance in the analysis, the highest dDDH score was 59.4 % against *S. infantis* UC6950A, which is also below the established 70 % threshold for within-species classification.

## 4 Discussion

Here, we present an as-yet undescribed *Streptococcus* species with unusual carbapenem resistance. The isolate was obtained from blood culture of a patient with severe underlying conditions who suffered from a polymicrobial bloodstream infection that resulted in a fatal outcome. Due to unusual phenotypic resistance to all beta-lactams tested, further phenotypic and genotypic characterization of the strain was performed.

The use of conventional phenotypic methods to identify alpha-haemolytic streptococci in general [30] and viridans streptococci such as Mitis streptococci in particular, has previously been shown to have limited utility [39,40]. MALDI-ToF MS also exhibits suboptimal performance in distinguishing between species within the *S. mitis*-group [39,40]. This limitation is evident in the case of SO-23-1, as this strain was misidentified as *Streptococcus mitis/oralis*. SO-23-1 exhibited anticipated growth patterns when cultivated in Tryptic Soy Broth supplemented with various concentrations of NaCl, up to a maximum of 4 %. The NaCl-test serves as a means of distinguishing between Streptococci and Enterococci. Streptococci, in contrast to Enterococci, fail to thrive in NaCl concentrations of 6.5 % or above.

SO-23-1 was classified as *Streptococcus intermedius* with a 91.20 % similarity (<80 % for API 20 Strep is considered unacceptable for identification), as determined through the analysis of the API 20 STREP test. However, due to the test's limited efficacy in accurately distinguishing between strains within the *S. mitis* group [30], we did not rely on this result.

Genotypic characterization of the housekeeping gene 16S rRNA revealed 98.1–99.2 % sequence similarity with *S. oralis* type strains, with the highest similarity to *S. oralis* subsp. dentisani 7747T. However, it has been established that the 16S rRNA gene is insufficient for discrimination between closely related species within the *Streptococcus* genus. New bioinformatics techniques, such as dDDH and ANI, offer better accuracy in discriminating between closely related species. Using these tools and relevant databases, we were unable to identity other Streptococci as above threshold for within-species classification, indicating that strain SO-23-1 was a novel species.

Genes encoding beta-lactam antibiotic resistance in *Streptococci* is uncommon, but mutations that alter the penicillin-binding proteins (PBPs), the targets for all β-lactam drugs, have been previously described [32]. Although decreased susceptibility against penicillins and cephalosporins is not uncommon in VGS, carbapenem antibiotic resistance is still rare [41,42]. Carbapenemase antibiotic resistance in VGS represents a concerning healthcare issue due to its impact on patient treatment. The standard treatment of severe infections, such as infective endocarditis and septicemia, caused by *Streptococci* in Norway is benzylpenicillin, possibly in combination with gentamicin. Here, several weeks of treatment with meropenem might have contributed to the mutations conferring carbapenem-resistance in the penicillin-binding proteins in strain SO-23-1. Alternatively, the strain could have possessed the variants of the penicillin-binding genes from the outset. Mosaic variants of such genes have been reported to be shared between various Streptococcal species [43]. Nevertheless, we believe that the highly unusual carbapenem resistance in strain SO-23–1 may have been a contributing factor to the patient's death due to delayed adequate antibiotic treatment.

In conclusion, combined results, including core genome phylogeny, protein homology, 16S similarity, ANI and dDDH suggest that strain SO-23-1 is indeed a novel species, which we designate *Streptococcus nidrosiense* (Latin adj.; pertaining to Nidaros, the historical name of the city of Trondheim, from where the strain was isolated).

## Ethical considerations

Ethical approval was obtained from the Norwegian Regional Committees for Medical and Health Research Ethics (REC), REC-number: 623773. This ethical approval includes a waiver of informed consent.

## CRediT authorship contribution statement

**Torunn Gresdal Rønning:** Writing – review & editing, Writing – original draft, Visualization, Validation, Software, Project administration, Methodology, Investigation, Funding acquisition, Formal analysis, Data curation, Conceptualization. **Camilla Olaisen:** Writing – review & editing, Writing – original draft, Visualization, Validation, Supervision, Software, Project administration, Methodology, Investigation, Funding acquisition, Formal analysis, Data curation, Conceptualization. **Christina Gabrielsen Ås:** Writing – review & editing, Writing – original draft, Visualization, Validation, Software, Methodology, Investigation, Formal analysis, Data curation, Conceptualization. **Jan Egil Afset:** Writing – review & editing, Validation, Project administration, Methodology, Investigation, Conceptualization. **Maria Schei Haugan:** Writing – review & editing, Writing – original draft, Visualization, Validation, Supervision, Resources, Project administration, Methodology, Investigation, Funding acquisition, Formal analysis, Data curation, Conceptualization.

## Declaration of competing interest

There are no conflict of interest for any of the authors.

This project was supported by internal funds from the Clinic of Laboratory Medicine, St. Olavs Hospital, Trondheim University Hospital, Trondheim, Norway.
